# The lung cancer-associated blood biomarker hPG_80_ exhibits a reversible increase in response to smoking in asymptomatic individuals

**DOI:** 10.1186/s40364-025-00861-4

**Published:** 2025-11-13

**Authors:** Bérengère Vire, Léa Payen, Claire Vignault, Véronique Hofman, Charles Hugo Marquette, Jean-Philippe Berthet, Jacques Boutros, Marius Ilie, Guillaume Penaranda, Philippe Pourquier, Nassima Mimoun, Dominique Joubert, Alexandre Prieur, Paul Hofman

**Affiliations:** 1 Progastrin Manufacturing, Grabels, France; 2 Institute of Pharmaceutical and Biological Sciences (ISPB), Claude Bernard Lyon I, Lyon, France; 3 Department of Biochemistry and Molecular Biology, Lyon-Sud Hospital, Hospices Civils de Lyon, Pierre-Bénite, France; 4 Center for Innovation in Cancerology of Lyon (CICLY) EA 3738, Faculty of Medicine and Maieutic Lyon Sud, Claude Bernard University Lyon I, Oullins, France; 5 Service Recherche Clinique Et Innovation, Laboratoire Biogroup, Besançon, France; 6 IHU respirERA, Laboratoire de Pathologie Clinique Et Expérimentale, Centre Hospitalo-Universitaire de Nice (CHUN), FHU oncoage, IRCAN Inserm U1081, BB-0033-00025, Université Côte d’Azur, Nice, France; 7 IHU respirERA, Service de Pneumologie Et d’Oncologie Thoracique, Centre Hospitalo-Universitaire de Nice (CHUN), FHU oncoage, Université Côte d’Azur, Nice, France; 8 IHU respirERA, Service de Chirurgie Thoracique, Centre Hospitalo-Universitaire de Nice (CHUN), FHU oncoage, Université Côte d’Azur, Nice, France; 9 Biogroup Provence, Marseille, France; 10 European Liquid Biopsy Society, Martinistrasse, Hamburg, Germany

## Abstract

The blood biomarker hPG_80_ is linked to multiple solid tumors, including lung cancer. This study examined blood hPG_80_ levels of asymptomatic individuals and patients with non-small cell lung cancer (NSCLC), categorized by their smoking and chronic obstructive pulmonary disease (COPD) status. Plasma hPG_80_ levels were measured across five cohorts of patients, including 396 NSCLC patients, 200 NSCLC cancer-free COPD patients, 369 asymptomatic never smokers, 278 asymptomatic current smokers, and 235 asymptomatic former smokers. Receiver operating characteristic (ROC) curves assessed diagnostic accuracy. In asymptomatic current smokers, hPG_80_ levels were significantly higher (6.70 pM (IQR: 5.13–11.29)) than those in gender- and age-matched never smokers (2.50 pM (IQR: 1.70–3.70; *p* < 0.0001). In contrast, gender- and age-matched former smokers showed a return to normal hPG_80_ levels (2.29 pM (IQR: 1.61–2.97)). In multivariate analysis, age and smoking status were significantly associated with elevated levels of hPG_80_ *(p*-values of 0.0319 and < 0.0001, respectively). Levels of hPG_80_ in current smokers were not different from levels found in age-matched patients with NSCLC or COPD (6.60 pM (IQR: 4.36–11.22) and 6.07 pM (IQR: 3.99–11.69), respectively). In NSCLC and COPD patients, hPG_80_ levels were independent of the smoking status. When comparing asymptomatic and NSCLC-diagnosed former smokers, the AUC was 0.85 (95% CI:0.80–0.90, *p* < 0.0001). The AUC was equal to 0.53 (95% CI: 0.45–0.60, *p* = 0.4436) for current smokers. Our findings identify hPG_80_ as both a reversible marker of active smoking and a diagnostic biomarker of NSCLC. This dual role supports its potential use in risk stratification and early detection, particularly among non-COPD former smokers.

**Supplementary Information:**

The online version contains supplementary material available at 10.1186/s40364-025-00861-4.

## To the Editor

Lung cancer is the leading cause of cancer death (GLOBOCAN 2022) [1], with smoking as the main risk factor for NSCLC [2]. While Low-Dose Computed Tomography (LD-CT) improves early detection, it lacks specificity [3]. Blood biomarkers like CEA, CYFRA21-1, and SCC-Ag are less invasive but have limited sensitivity for early-stage NSCLC detection [4].

hPG_80_ (circulating progastrin) is a promising blood biomarker for detection of solid tumors [5]. Activation of oncogenic pathways (APC/β-catenin, Ras) drives secretion of hPG_80_ in cancer cells, where it promotes tumorigenic processes including cancer stem cell survival [6]. Previous work has shown elevated hPG_80_ in NSCLC [5, 7] but it remains unknown whether tobacco exposure can influence hPG_80_ and whether such changes are reversible. hPG_80_ levels were also measured in patients with COPD, a common smoking-related condition not considered a confounding factor of tobacco consumption [8].

Plasma hPG_80_ was measured in five cohorts: treatment-naïve NSCLC patients (*n* = 396), COPD patients without NSCLC (*n* = 200), and asymptomatic never (*n* = 369), current (*n* = 278), and former smokers (*n* = 235) (Table S1 and Supplementary methods). Quantification was performed using the DxPG80.lab ELISA [9], with gender- and age-matched comparisons. Multivariate analysis was performed to assess the effects of gender, age, pack-years, and smoking history on hPG_80_ variations (Supplementary methods).

### Smoking induces an increase in hPG_80_ blood levels in asymptomatic individuals

**Fig. 1:**
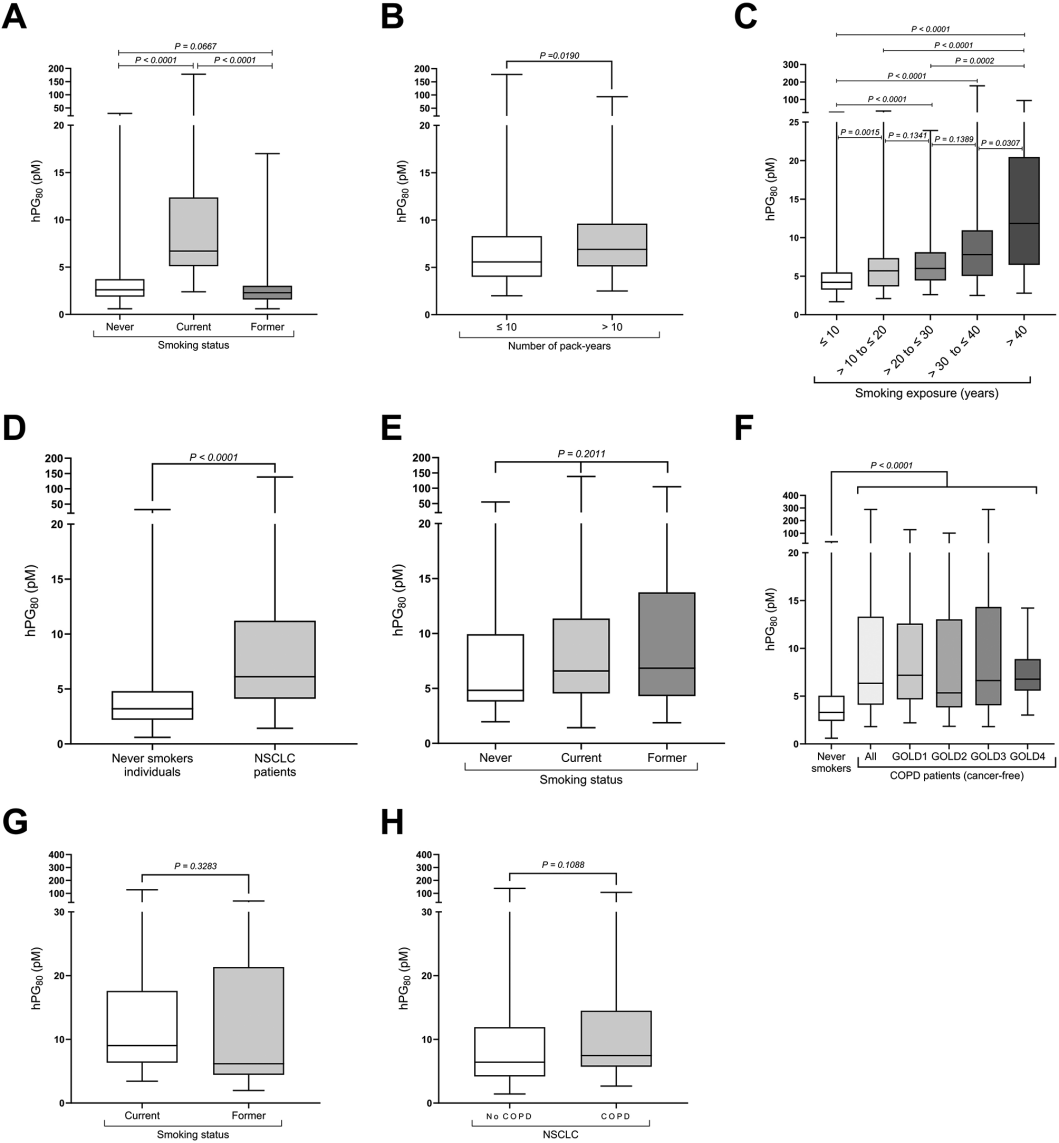
Impact of smoking, NSCLC and COPD on hPG_80_ levels. **A**. Comparison between gender- and age-matched current smokers, never smokers and former smokers (*n* = 110). **B**. hPG_80_ levels in current smokers (*n* = 83) stratified according to the number of pack-years (≤10 and > 10). **C**. Incidence of the number of years of smoking exposure on hPG_80_ levels in current smokers. **D**. Comparison of hPG_80_ levels between age-matched never smokers and NSCLC patients (*n* = 289). **E**. hPG_80_ levels stratified according to the smoking status: never (*n* = 40), current (*n* = 224) and former (*n* = 127). **F**. Comparison of age-matched never smokers and COPD patients (*n* = 145), stratified according to the gold status (GOLD1, *n* = 40; GOLD2, *n* = 60; GOLD3, *n* = 39; and GOLD4, *n* = 6) or pooled (all stages). **G**. hPG_80_ levels in cancer-free COPD patients according to the smoking status: current (*n* = 27) and former (*n* = 18). **H**. hPG_80_ levels in NSCLC patients stratified according to the COPD status: no COPD (*n* = 364) and COPD (*n* = 32). Boxes represent the interquartile range, and the horizontal line across each box indicates median values. The statistical differences were evaluated using the Kruskall-Wallis test and the Mann-Whitney *U* test

Active smoking was strongly associated with elevated hPG_80_ in asymptomatic individuals: median 6.70 pM (IQR: 5.13–11.29) versus 2.50 pM (IQR: 1.70–3.70) in gender- and age-matched never smokers (*p* < 0.0001). Levels correlated to smoking duration and cumulative exposure (Fig. 1A–C and Table S2). In multivariate analysis, smoking status and age were independently associated with higher circulating hPG_80_ levels (Table S3 and Fig. S1). Strikingly, former smokers without NSCLC had hPG_80_ levels similar to never smokers (median 2.29 pM (IQR: 1.61–2.97)) (Fig. 1Aand Table S2). Notably, 82% of former smokers had hPG_80_ levels below the limit of quantification (LoQ). hPG_80_ levels drop within the first year after quitting smoking (low in 88% of recent quitters), suggesting that the elevation in smokers is reversible (Fig. S2). The effect size (4.41; 95% CI: 3.94–5.45; *p* < 0.0001) further supports the robustness of these findings (Table S4A).

### hPG_80_ levels in NSCLC and COPD patients

In NSCLC patients, hPG_80_ was significantly higher than in age-matched asymptomatic never smokers (6.11 pM (IQR: 4.11–11.22) versus 3.20 pM (IQR: 2.20–4.80); *p* < 0.0001) (Fig. 1D), regardless of the stage or histology (Fig. S3), and independent of the smoking status (Fig. 1E). COPD patients without NSCLC also displayed elevated hPG_80_ (6.35 pM (IQR: 4.10–13.31) versus 3.30 pM (IQR: 2.40–5.05) in age-matched asymptomatic never smokers; *p* < 0.0001) (Table S2 and Fig. 1F). The hPG_80_ increase in COPD patients was independent of disease severity, smoking status, or the presence of NSCLC (Fig. 1F–H).

### hPG_80_ levels in NSCLC compared to their respective self-declared cancer free age-matched controls

**Fig. 2:**
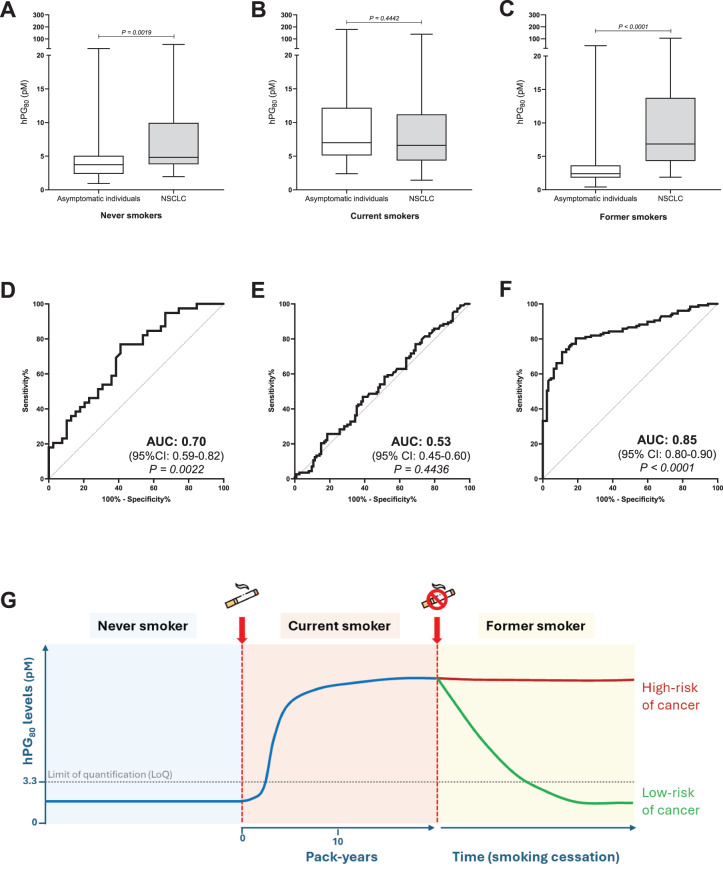
hPG_80_ in former smokers: strong diagnostic value and potential marker of residual cancer risk. Comparison of hPG_80_ levels in NSCLC patients versus their respective age-matched controls, stratified according to the smoking status: **A**. never (*n* = 39), **B**. current (*n* = 113) and **C**. former smokers (*n* = 127). Boxes represent the interquartile range, and the horizontal line across each box indicates median values. The statistical differences were evaluated with the Mann-Whitney *U* test. ROC curve analyses corresponding to each comparison: **D**. never, **E**. current and **F**. former smokers. AUC: area under the curve. **G**. Hypothetical link between the hPG_80_ levels and risk of cancer in former smokers. hPG_80_ levels are low in never smokers, rise with smoking exposure up to 10 pack-years, and may decrease after cessation in some individuals, while persistently elevated levels could indicate a higher risk of cancer

When NSCLC patients were compared to their respective controls matched for smoking status, the levels of hPG_80_ were significantly higher in never and former smokers, but not different from current smokers (Fig. 2A–C and Table S2). Effect size analysis confirms a clear median difference between NSCLC patients and former smokers (4.44; 95% CI: 3.38–5.36; *p* < 0.0001) (Table S4B). Diagnostic performance (AUC) was highest in former smokers (0.85 (95% CI = 0.80–0.90), *p* < 0.0001) and never smokers (0.70 (95% CI = 0.59–0.82), *p* = 0.0022), but negligible in current smokers (0.53 (95% CI = 0.45–0.60), *p* = 0.4436) (Fig. 2D–F). For former smokers, ROC analysis identified an optimal cutoff of 4 pM (Youden’s index), with 81% sensitivity and 80% specificity.

## Discussion

This study shows a strong link between hPG_80_ blood levels and smoking, suggesting its potential use as: i) a motivational tool for quitting (due to post-cessation decline), ii) a risk marker in former smokers without COPD (Fig. 2G); and iii) a broad cancer detection biomarker in high-risk groups, given smoking’s link to multiple tumor types.

Nicotine can activate the Wnt/β-catenin pathway in bronchial epithelial cells [10]. This may trigger hPG_80_ secretion *via* the Wnt/β-catenin pathway, which directly targets its gene promoter. Chronic smoking-related inflammation and pre-neoplastic changes could further drive hPG_80_ expression before cancer appears. Given hPG_80_’s role in cancer stem cell survival [11], its elevation may promote field carcinogenesis and contribute to the higher cancer risk in smokers.

This study holds some limitations, including the limited associated clinical data for asymptomatic individuals and the incomplete smoking history of COPD patients. In addition, no comparison with other biomarkers was performed.

In conclusion, our findings support a dual role for hPG_80_: as a biomarker of smoking-related biological risk and as a diagnostic biomarker for lung cancer. Therefore, hPG_80_ could become a valuable tool for early NSCLC detection in non-COPD former smokers, who represent 18% of the adult population [12], warranting prospective validation in screening programs.

## Electronic supplementary material

Below is the link to the electronic supplementary material.

Supplementary Material 1

## Data Availability

No datasets were generated or analysed during the current study.

## Abbreviations

AUC: Area under the curve
95% CI: 95% Confidence interval
COPD: Chronic obstructive pulmonary disease
IQR: Interquartile range
LD-CT: Low-dose computed tomography
LoQ: Limit of quantification
NSCLC: Non-small cell lung cancer
ROC: Receiver operating characteristic
