# Supplementary material for: The lung cancer-associated blood biomarker hPG80 exhibits a reversible increase in response to smoking in asymptomatic individuals
Source: Biomark Res. 2025 Nov 13;13:148. doi: 10.1186/s40364-025-00861-4 (PMC12613591; doi:10.1186/s40364-025-00861-4)
Supplement: Supplementary file 1 — Supplementary Material 1 [file 40364_2025_861_MOESM1_ESM.docx]

**Supplementary Materials**

**Supplementary Methods**

**Supplementary Figure S1***. Interaction curves of the multivariate models’ cofactors on age-matched cohorts.*

**Supplementary Figure S2.** *hPG_80_ levels in* *never, current and* *former smokers stratified by smoking cessation time.*

**Supplementary Figure S3.** *hPG_80_ levels in* *NSCLC patients according to disease stage and histological subtype.*

**Supplementary Table S1.** *Demographic and clinical characteristics of NSCLC patients (n=396), NSCLC-cancer free COPD patients (n=200), asymptomatic never smokers (n=369), current smokers (n=278) and former smokers (n=235).*

**Supplementary Table S2.** *Age and hPG_80_ levels in the different cohorts of asymptomatic individuals and patients.*

**Supplementary Table S3.** *Univariate and multivariate analyses of the effect of each parameter on hPG_80_ levels based on data matched for age, gender, cohort, pack per year, and pack per year cohort interaction.*

**Supplementary Table S4***. Effect size analyses within the cohort of asymptomatic individuals (A) and, between age-matched former smoker asymptomatic individuals and NSCLC patients (B).*

**Supplementary Methods**

*NSCLC and COPD patients*

EDTA plasma samples from treatment-naïve 396 NSCLC patients collected at diagnosis and from 200 COPD patients were obtained from the Nice Hospital Biobank (BB-0033-00025; Nice, France). All patients signed an informed consent form and the study was approved by the local research ethics committee (CHUN_DRCI_Nice_004-2022_Airproject).

*Asymptomatic never, current and former smoker’s individuals*

EDTA plasma samples were collectively prospected from 369 never smokers’ individuals, 278 current smokers and 235 former smokers. These samples were obtained from Biogroup laboratory, which reuses patient data and biological materials for research purposes. The subjects received an information letter regarding the Biogroup biological sample and associated data collection. Samples are anonymized, and only the associated data age group, sex, and smoking status are retained.

*hPG_80_ level measurements in the blood samples*

The ELISA DxPG80.lab kit (Biodena Care, Grabels, France) was used to measure hPG_80_ levels in plasma samples according to the manufacturer’s instructions described previously [1].

*Statistical analysis*

The distribution of hPG_80_ levels in the five patient cohorts was assessed for normality using a Shapiro-Wilk normality test. Results indicated that the distributions were not normal (*p* < 0.05 for all cohorts). Therefore, non-parametric tests were used for group comparisons. Statistical differences in hPG_80_ levels for comparisons involving more than two groups were evaluated using the non-parametric Kruskall-Wallis. When the global test indicated statistical significance, post hoc pairwise comparisons were subsequently performed between groups using the Mann-Whitney *U* test. Data are expressed as median ± interquartile range (IQR, Q1-Q3). Gender distribution was compared using the chi-square test. Effect sizes were assessed as differences in medians between groups, with 95% confidence intervals (95% CI) estimated via bootstrap resampling (1,000 iterations) using the percentile method. To minimize potential confounding due to age, asymptomatic donors were age-matched to NSCLC patients within each smoking status category (never smokers, former smokers, and current smokers). The matching was performed independently for each group using MedCalc Statistical Software version 23.2.1, employing a 1:1 nearest-neighbor matching algorithm without replacement. Gender was not considered in the matching process, as no significant differences in hPG_80_ levels were observed between males and females. The diagnostic discriminative accuracy of hPG_80_ levels in patients with NSCLC compared to asymptomatic donors was assessed using Receiver Operating Characteristics (ROC) curve analyses. The optimal cutoff value was determined using Youden’s index from the ROC curve analysis.

An additional propensity score matching approach was applied within asymptomatic smoking category sub-cohorts, based on age and gender. Triplets of observations were constructed by minimizing the Euclidean distance between propensity scores, each triplet comprising one observation from each cohort, with no subject duplication (each individual was included in only one triplet). A total of 235 unique triplets were generated. Among these, the 110 triplets with the lowest Euclidean distances were selected based on a threshold defined using the Elbow Method. The effectiveness of propensity score matching was evaluated by comparing age and sex distributions before and after matching and by calculating standardized mean differences (SMD) for these variables, with SMD values close to zero indicating excellent balance across all cohort comparisons. Univariate and multivariate analyses (generalized linear mixed models) were conducted to assess the effects of gender, age, pack-years, and smoking category sub-cohort on hPG_80_ variations. For qualitative variables, the median (Q1-Q3) hPG_80_ values were reported for each category, along with *p*-values; for the quantitative variable (age), the regression coefficient β (95% CI) from simple linear regression was reported. The regression coefficient β (95% CI) was reported for multivariate regression along with *p*-values. All calculations were performed using a significance threshold of alpha = 0.05. Statistical analyses were carried out using MedCalc Statistical Software version 23.2.1, Prism software (GraphPad Prism version 9.4), and SAS V9.4 (SAS Institute Inc., Cary, NC, USA).

**Supplementary FIGURE S1**. *Interaction curves of the multivariate models' cofactors on age-matched cohorts*. Vertical axis scale was shortened to 0–30 pM, which excludes six data points above this range.


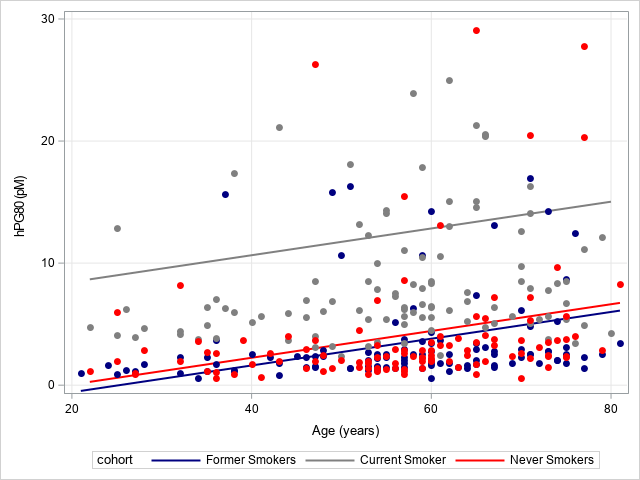


**Supplementary FIGURE S2**. *hPG_80_* *levels in* *never, current and* *former smokers stratified by smoking cessation time.* Smoking cessation time categories (years): <1 (n=17), 1-2 (n=9), 3-5 (n=16), 6-10 (n=39), 11-20 (n=48), 20-30 (n=42) and >30 (n=64). Boxes represent the interquartile range, and the horizontal line across each box indicates median values. The statistical differences were evaluated using the Mann-Whitney *U* test.

**Supplementary FIGURE S3**. *hPG_80_ levels in* *NSCLC patients according to disease stage and histological subtype.* **A**. hPG_80_ levels by disease stage: stage I (n=100), stage II (n=100), stage III (n=97) and stage IV (n=99). **B**. hPG_80_ levels in subgroups of NSCLC characterized by histological subtypes. LADC: adenocarcinoma, LSCC: squamous cell carcinoma, LLCC: large cell carcinoma. Boxes represent the interquartile range, and the horizontal line across each box indicates median values. The statistical differences were evaluated using the Mann-Whitney *U* test (A) and Kruskall-Wallis test (B).

**B**

**A**

**Supplementary Table S1**. *Demographic and clinical characteristics of NSCLC patients (n=396), lung-cancer free COPD patients (n=200), asymptomatic never smokers (n=369), current smokers (n=278) and former smokers (n=235).*

Abbreviations: COPD, chronic obstructive pulmonary disease; SD, standard deviation, GOLD, Global Initiative for Chronic Obstructive Lung Disease

**Supplementary** **Table S2**. *Age and hPG_80_ levels in the different cohorts of asymptomatic individuals and patients.*


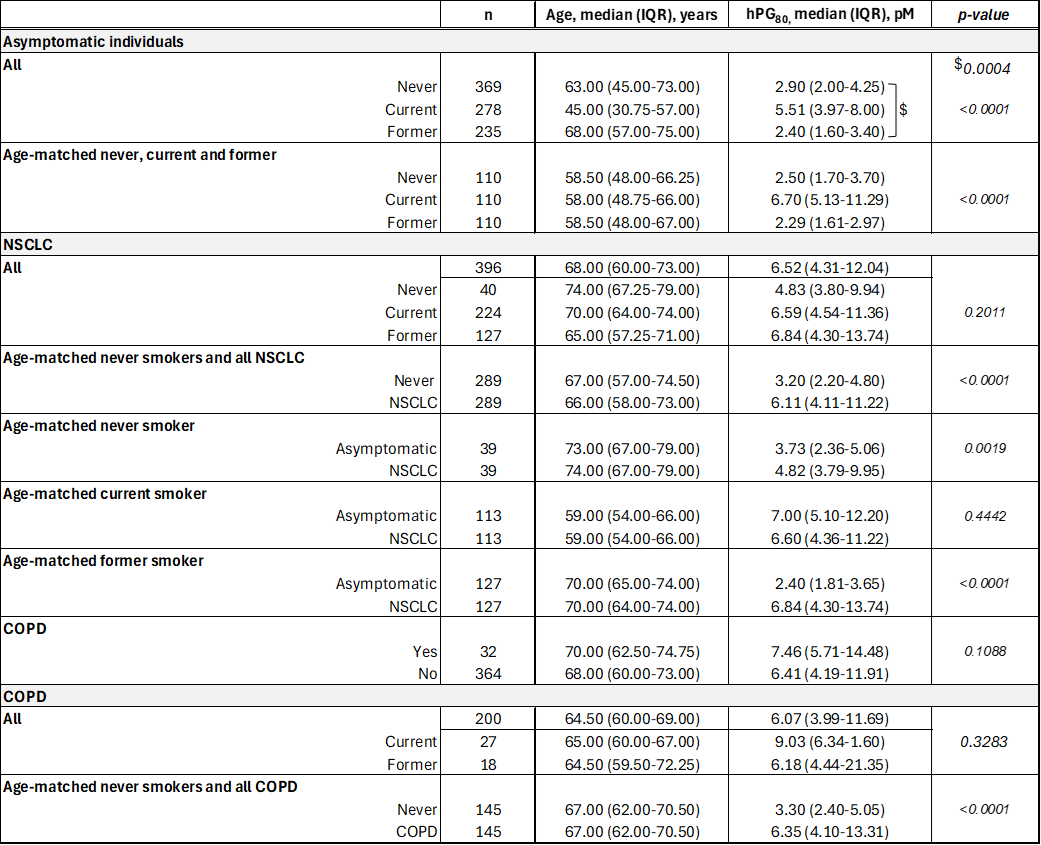


Abbreviations: NSCLC: non-small cell lung cancer; COPD, chronic obstructive pulmonary disease; IQR: interquartile range.

**Supplementary Table S3**. *Univariate and multivariate analyses of the effect of each parameter on hPG_80_ levels based on data matched for age, gender, cohort, pack per year, and pack per year cohort interaction.*

| **Parameters** |  | **Univariate** |  | **Multivariate** | |
| --- | --- | --- | --- | --- | --- |
|  | **n** | **hPG_80_, median (q1q3) / β (95%CI), pM** | ***p-value*** | **β (95%CI)** | ***Overall p-value*** |
| **Gender** |  |  | *0.4603* |  | *0.5984* |
| Female | 147 | 3.50 (2.00-6.17) |  | 0.76 (-2.07-3.58) |  |
| Male | 183 | 3.24 (2.00-7.32) |  | Reference |  |
| **Age** | 330 | 0.11 (0.01-0.21) | *0.0376* | 0.11 (0.01-0.21) | *0.0319* |
| **Cohort** |  |  | *<0.0001* |  | *<0.0001* |
| Former smokers | 110 | 2.29 (1.61-2.97) |  | -0.99 (-4.56-2.58) |  |
| Current smokers | 110 | 6.70 (5.13-11.29) |  | 7.98 (4.30-11.65) |  |
| Never smokers | 110 | 2.50 (1.70-3.70) |  | Reference |  |
| **Pack-years** |  |  | *0.0380* |  | *0.6032* |
| 0 | 110 | 2.50 (1.70-3.70) |  | Reference |  |
| < 10 | 92 | 4.15 (2.29-6.37) |  | 0.93 (-2.59-4.45) |  |
| ⩾ 10 | 128 | 5.12 (2.25-8.52) |  | 0 |  |

Univariate results are expressed as follows: (i) for qualitative variables, median (q1q3) of hPG_80_ in each category, with p-values; (ii) for the quantitative variable (age), as regression coefficient β (95% CI) from simple linear regression. Multivariate results are expressed as regression coefficient β (95% CI) for multiple linear regression. The “Overall *p*-value” column reflects the overall significance of each variable in a multiple linear regression model.

**Supplementary Table S4**. *Effect size analyses within the cohort of asymptomatic individuals (A) and, between age-matched former smoker asymptomatic individuals and NSCLC patients (B).*

**A**

| **Age-matched never, current and former smoker** | **N** | **Age** | **hPG_80,_ median (q1q3), pM** | **Median Difference (95%CI)***  **Current - Never** | **Median Difference (95%CI)***  **Former- Never** | **Median Difference (95%CI)***  **Current-Former** | ***p*** |
| --- | --- | --- | --- | --- | --- | --- | --- |
| Never | 110 | 58.50 (48.00-66.25) | 2.50 (1.70-3.70) | 4.20 (3.65 - 5.19) | -0.21 (-0.61 - 0.004) |  | *<0.0001* |
| Current | 110 | 58.00 (48.75-66.00) | 6.70 (5.13-11.29) |  |  | 4.41 (3.94 - 5.45) |  |
| Former | 110 | 58.50 (48.00-67.00) | 2.29 (1.61-2.97) |  |  |  |  |

*Difference between current smokers and never smokers medians, between former smokers and never smokers medians, and between former and never smokers medians; 95%CI were obtained via bootstrap resampling (1,000 iterations) using the percentile method.

**B**

| **Age-matched former smoker** | **N** | **Age** | **hPG_80,_ median (q1q3), pM** | **Median Difference (95%CI)*** | ***p*** |
| --- | --- | --- | --- | --- | --- |
| Asymptomatic | 127 | 70.00 (65.00-74.00) | 2.40 (1.81-3.65) | 4.44 (3.38 - 5.36) | *<0.0001* |
| NSCLC | 127 | 70.00 (64.00-74.00) | 6.84 (4.30-13.74) |  |  |

*Difference between NSCLC and asymptomatic medians; 95%CI were obtained via bootstrap resampling (1,000 iterations) using the percentile method.

**REFERENCES**

1. Cappellini M, Flaceliere M, Saywell V, Soule J, Blanc E, Belouin F, et al. A novel method to detect hPG80 (human circulating progastrin) in the blood. Anal Methods-uk. 2021;13:4468–77.
